# Assessment of Content and Release of Nickel, Cobalt and Lead in Metallic and Rhinestone Nail Charms in the United States

**DOI:** 10.1111/cod.70233

**Published:** 2026-08-20

**Authors:** Emily Ames, Tarah Schaffer, Brianna C. Peacock, David Cotter, Dathan Hamann, Carsten R. Hamann

**Affiliations:** ^1^ Kirk Kerkorian School of Medicine at UNLV Las Vegas Nevada USA; ^2^ Saguaro Dermatology Phoenix Arizona USA; ^3^ The University of Arizona College of Medicine Phoenix Phoenix Arizona USA; ^4^ HonorHealth Dermatology Residency Scottsdale Arizona USA; ^5^ Arizona State University John Shufeldt School of Medicine and Medical Engineering Phoenix Arizona USA; ^6^ Las Vegas Dermatology Las Vegas Nevada USA

**Keywords:** allergic contact dermatitis, cobalt, dimethylglyoxime test, metal allergy, nail charms, nickel

## Abstract

**Background:**

Metallic nail adornments and other charms are increasingly popular additions to traditional nail treatments. Nickel and cobalt are well‐recognised causes of allergic contact dermatitis (ACD) in jewellery and other daily consumer exposures. Nickel‐releasing nail charms complicating acrylate allergic contact dermatitis in a young woman have been reported. No current studies have evaluated the presence of nickel and cobalt in metallic nail charm products in the United States.

**Objective:**

To assess nickel and cobalt release and total metal content with x‐ray fluorescence spectroscopy (XRF) from nail charm products available in the United States.

**Methods:**

Convenience samples of nail charm products were obtained from nail supply stores in Arizona, Nevada and online retailers. Nickel and cobalt content and release were assessed in 231 nail charms from 25 nail product brands by spot tests and XRF, respectively. In charms with lead content assessed by XRF, confirmatory spot testing was performed.

**Results:**

Nickel release was detected in 89/231 (38.5%) products by dimethylglyoxime (DMG) spot test and 156/231 (67.5%) products by XRF. Cobalt release was not detected. Lead was detected in 5/231 (2.2%) products by XRF and 2/231 (0.87%) by spot test.

**Conclusions:**

Nickel release is common from nail charm products purchased in the United States, which could be an emerging novel source of consumer and occupational nickel exposure. Lead was also detected in a small subset of charms, indicating that nail charms may be a rare but emerging source of exposure to lead.

## Graphical abstract

Nail charms are increasingly popular additions to traditional nail treatments. Nickel release was detected in 38.5% of nail charm products by DMG spot test and 67.5% of products by XRF. Nail charms may represent an underrecognized source of consumer exposure in the United States.

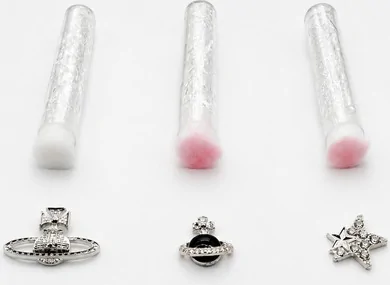

**Abbreviations**
ACD: allergic contact dermatitis
DMG: dimethylglyoxime
ICP‐MS: inductively coupled plasma mass spectrometry
XRF: X‐ray fluorescence spectroscopy

## 1 Introduction

Nickel was named “Allergen of the Year” by the American Contact Dermatitis Society in 2008 [1]. Nickel and cobalt are well‐recognised causes of allergic contact dermatitis (ACD) in jewellery, belts and other daily exposures [2, 3, 4]. A recent case report described nickel‐releasing nail charms complicating acrylate allergic Contact Dermatitis in a young woman [5]. Although nickel content in nail charms has been assessed in Korea [6], no studies have evaluated the presence of nickel and cobalt in metallic nail charm products in the United States.

Skin piercings and jewellery are leading exposures for nickel sensitization [1]. A prior Korean study evaluated nail stickers, tips and charms for nickel, cobalt and chromium: spot testing identified nickel release in 8.1% of the nail products tested [6]. Following complete microwave processing with nitric acid and hydrofluoric acid, inductively coupled plasma mass spectrometry (ICP‐MS) detected nickel, cobalt and chromium in 11%, 6.3% and 16.7% of the samples, respectively [6]. Overall, the study found detection rates to be highest in nail products with rhinestones [6].

## 2 Materials and Methods

A total of 231 convenience samples were collected from 25 nail beauty product brands in the United States. Five of these brands were purchased from beauty supply stores in Las Vegas, Nevada, 5 were purchased in Mesa, Arizona and 15 were purchased from online retailers. The brands included *Après, FP Fingerpaints, Jia Yi, Jewellery Nail, JelloJello, make.N, Zoklu, Teenitor, CnSybaris, Quezadyl, Ganepwns, Belicey, Sutokaka, iOhauu, Sutozmiwe and Itpoyol*. Some of the nail products purchased in Las Vegas, NV (i.e., NV Nail & Beauty Supply, Direct Beauty Supply), Mesa, AZ (i.e., Nationwide Beauty Supply) and from online retailers (i.e., The Nail Superstore, Universal Nail Supplies) were unbranded.

The nail products purchased were metallic charms, rhinestone‐containing charms, or both. Rhinestones are usually made from glass, crystal, or synthetic polymers like acrylic. It was not possible to characterise their material. Based on visual inspection, 147 were made of metal, 17 were made of rhinestones and 67 were made of both rhinestones and metals. The nail charms varied in size and shape, but the majority were small (< 1 cm) and were shaped as crosses, bows, flowers, or circular rhinestones.

Qualitative nickel and cobalt spot tests were conducted on all nail charms to determine if there was nickel or cobalt release, respectively. The Reveal&Conceal (*SmartPractice*, *Phoenix, AZ*) spot test, which is a quick liquid swab test that uses dimethylglyoxime (DMG), which chemically reacts with free nickel, was used to spot test for the presence of nickel. A positive test resulted when the applicator turned pink (Figure 1). Negative tests were indicated by no colour change. The Reveal&Conceal (*SmartPractice*, *Phoenix, AZ*) Cobalt spot test which is a liquid swab test filled with a disodium‐1‐nitroso‐2‐naphthol‐3,6‐disulfonate solution was used to detect the presence of cobalt in the nail charms. A positive test resulted when the applicator turned pink, orange, or red. Charms were excluded if they were circular rhinestone charms smaller than 4 mm or metal charms smaller than 1 mm because it was not feasible to perform the spot test with these charms.

**FIGURE 1:**
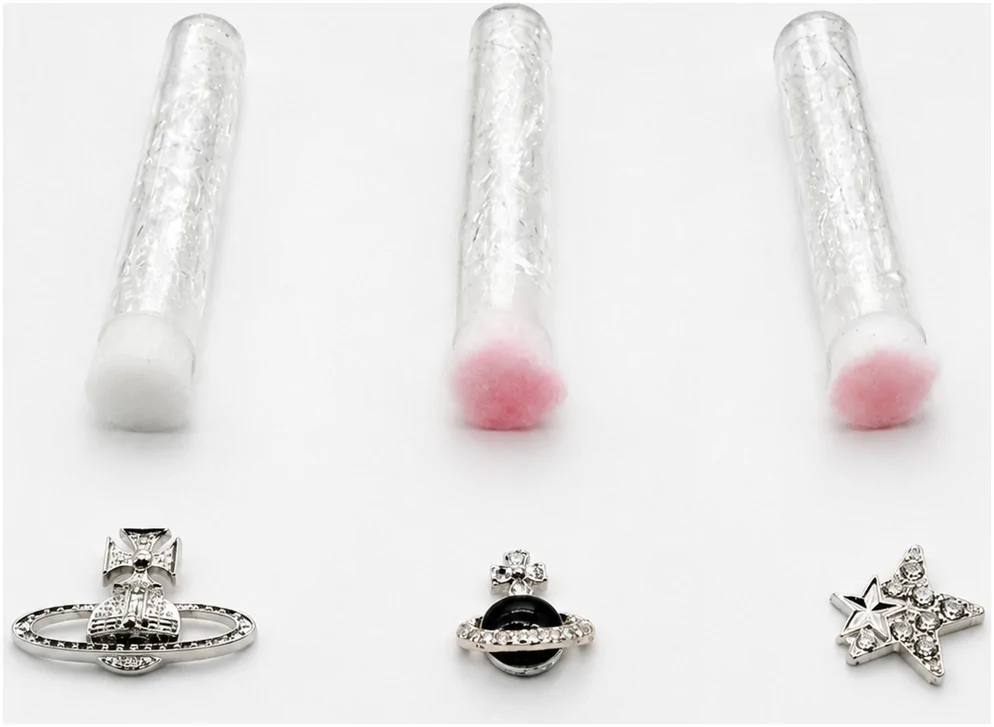
DMG spot testing demonstrating negative (left) and positive (middle and right) results for nickel release.

Quantitative x‐ray fluorescence spectroscopy (XRF) was performed with a *Bruker S1 Sorter* (Billerica, Massachusetts, USA; Software Version is 1.0.3.0) to assess the presence of nickel, cobalt and lead. Analysis on the Bruker S1 Sorter was set with a range of 3–10 s intervals, consistent with the standard protocol for this instrument and previous studies [2]. The presence of nickel, cobalt and the two metals making up the largest percentage of the charm were recorded. Nickel and cobalt were recorded if they were > 1% on XRF. If lead was present, the amount was also recorded. If a charm was negative for nickel or cobalt on the swab test, but positive on XRF, the charm was retested with spot testing and XRF.

If a charm was positive for lead on XRF, a lead swab test was performed using the *NewPos* Lead Swab Test. A positive swab was indicated by a purple colour change and a negative swab was yellow (Figure 2). If a charm was positive for lead on XRF, but negative on the swab test, the charm's front (paint portion, if applicable) was also tested.

**FIGURE 2:**
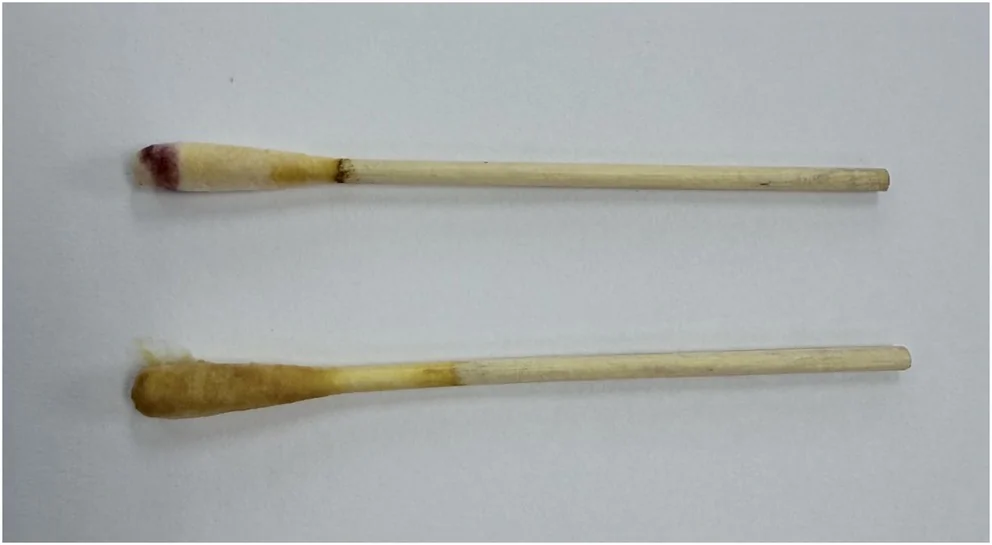
Positive (top) and negative (bottom) lead test swabs. Purple coloration (top) signifies positive result.

## 3 Results

Nickel release was detected in 89/231 (38.5%) charms. No charms released cobalt as assessed by spot test. Of the 89 charms that released nickel, 51/89 (57.3%) were metal, 0/89 (0%) were rhinestones and 38/89 (42.7%) were a mixture of both.

XRF detected nickel in 156/231 (67.5%) nail charms. The level ranged from 1.03% to 48.9%. Of the charms positive for nickel on XRF, 101/231 (43.7%) were metal charms, 0/231 (0%) were rhinestone charms and 55/231 (23.8%) charms were a mixture of both metal and rhinestones. The highest level (48.9%) was from a metal charm purchased online from the brand *make.N*. XRF detected cobalt in 0 products.

The metals most identified by XRF were copper and zinc. Copper was the predominant component in 205/231 (88.7%) nail charms and zinc was the second most common component in 115/231 (49.8%) nail charms (Table 1). Of the 231 nail charms, 118/231 (51.1%) were brass (copper and zinc) alloys.

**TABLE 1:** Distribution of the most common and second most common components in nail charms, identified by XRF.

Metals	Most common component^a^ (*n*)	Second most common component^b^ (*n*)
Cu	205	13
Fe	10	5
Ti	7	5
Zn	4	115
Ni		70
Cr		8
Sb		7
Pb		6
Sn		1
V		1
W		1

^a^ Count rate too low for one charm (*n* = 1).

^b^ *n* exceeds 213 because one charm contained equal amounts of Fe and Pb for the second most common component.

Lead was identified by XRF in 5/231 (2.2%) nail charms. Swab tests were positive for lead release in 2 of these 5 charms (Figure 3). XRF levels were 3.3%, 5.84%, 9.49%, 9.79% and 17.81%. The highest level (17.81%) was from a metal charm with an unidentified brand purchased from the store NV Nail & Beauty Supply in Las Vegas, NV.

**FIGURE 3:**
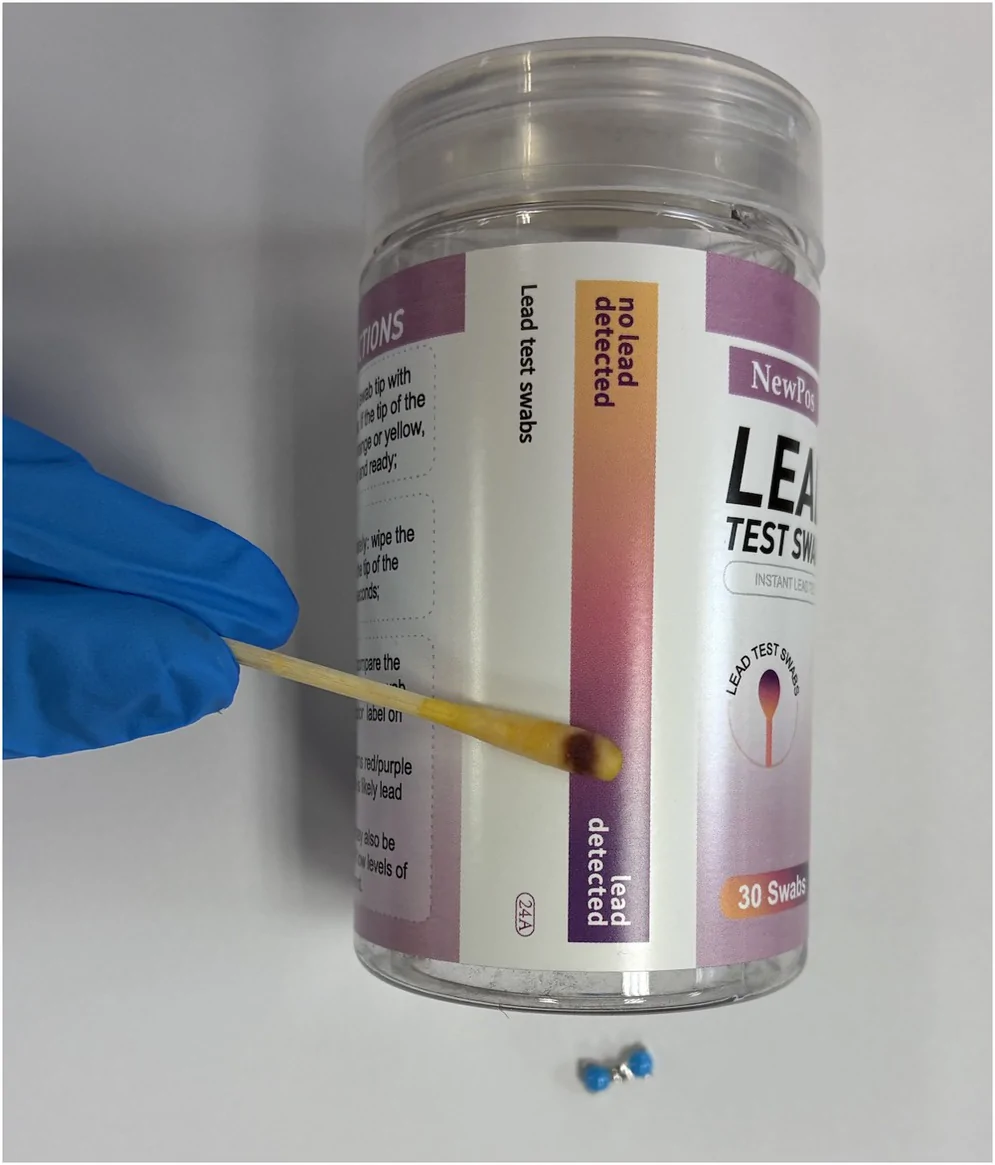
Example of a nail charm positive for lead with lead swab.

The mean, median and mode costs of each charm were $0.15/charm, $0.07/charm and $0.01/charm, respectively.

## 4 Discussion

Nickel content and release is common in modern nail charms available in the United States. While methylacrylates, which are found in artificially sculpted nails, have been described as allergens involved in irritant and allergic contact dermatitis, little is known about metal allergen exposure from decorative nail adornment products [7, 8]. A retrospective analysis of North American Contact Dermatitis Group data found that 2% of patients had more than one allergic patch test reaction associated with a nail care product or an irritant Contact Dermatitis associated with a nail care product, with the hands affected in 27.6% of patients [9]. As nail cosmetics have evolved from simple manicures to more advanced procedures that can include nail adornments, these products may represent an overlooked source of nickel exposure for sensitised individuals (Figure 4) [10, 11].

**FIGURE 4:**
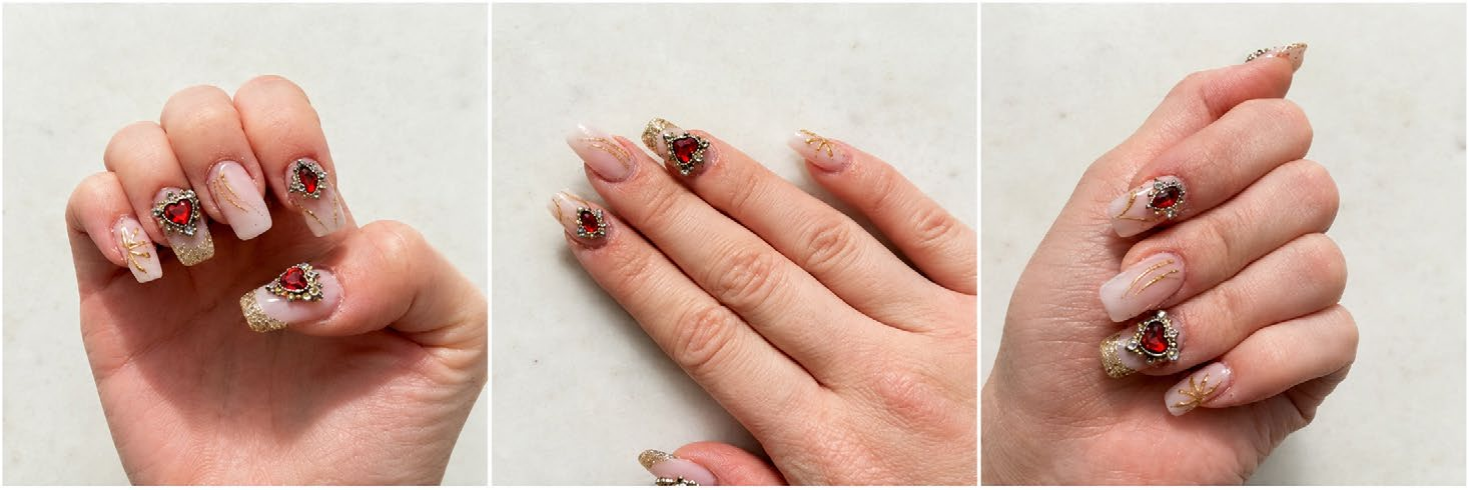
Example of nail adornments (mixture of metallic and rhinestone nail charms).

The rate of nickel release detected in our study was higher than expected. Kim et al.'s study identified nickel in 8.1% of nail products by spot testing and 11% by ICP‐MS [6]. Our findings were substantially higher and may represent a new or possibly an emerging source of consumer nickel exposure. The higher prevalence observed in our study could also be a result of differences in products available in the United States compared with world markets, as the United States lacks nickel legislation for consumer protection. Nickel‐containing charms may be particularly relevant when a patient presents with onycholysis and subungual hyperkeratosis, suggesting fingertip dermatitis from a possible contact allergy [12]. A recent case report described a patient with patch test‐confirmed nickel allergy who presented with recurrent dermatitis of her ears, eyelids, lips and fingers. DMG testing of her acrylic nail set decorated with metallic nail charms demonstrated nickel release [5].

Cobalt is a common allergic contact allergen and may co‐react with nickel during patch testing [13]. Since the EU Nickel Directive was passed, there has been postulation that more cobalt will be used in inexpensive jewellery products to replace the use of nickel [14]. We found 0 nail products to contain cobalt by x‐ray fluorescence spectroscopy. This was not surprising—despite speculation that the presence of cobalt would increase in jewellery, only one case report of dermatitis from cobalt found in jewellery products has been reported [15]. This may also reflect differences in industrial application of nickel and cobalt in jewellery products since nickel is commonly used as a pre‐plating or thin film material, whereas cobalt is less commonly used for this purpose.

Identification of lead content was not a primary goal of this study; there are no reports of lead in nail charm products and no cases of lead toxicity from a nail charm product. Nevertheless, we were surprised to find lead content by XRF in 2.2% or 5 products and this was further evaluated by spot test showing lead release in 2 of the products. Lead is a well‐established toxic metal and lead toxicity has adverse effects on multiple organ systems, including the neurologic, hematologic, cardiovascular, renal and reproductive systems [16]. Lead in nail charms may also represent an overlooked source of lead exposure for consumers and this likely warrants more investigation.

Kim et al.'s study found the highest rate of metal release to be in rhinestone products, which are not traditionally thought to be sources of metal contact allergens [6]. Interestingly, we did not have nickel release from any of our rhinestone products tested. This could be explained by manufacturing differences, including dyes used in rhinestones, product selection by country, or time‐dependent metal release not captured by spot testing.

Our study has several limitations. Since convenience samples were used, the nail charm products tested may not be representative of all nail charms available in the United States. Although nail charms were both qualitatively and quantitatively assessed for the presence of nickel and cobalt, there were no patients or follow‐up patch testing in our study, so the clinical relevance of our results cannot be assessed.

## 5 Conclusion

This study expands on Kim et al.'s study in Korea, which detected nickel, cobalt and chromium in nail tips and stickers [6]. The identification of nickel in nail charm products sold in the United States suggests that these metals are found in nail products outside of Korea and could represent a potential underrecognized source of metal allergen exposure worldwide.

The high frequency of nickel release observed in our study may have increasing importance given the global nail care products market is estimated to increase from 25.1 billion USD in 2025 to 37.2 billion USD in 2032, with nail enhancements representing the fastest growing market segment [17]. Furthermore, North America holds the largest share of the nail cosmetics market (25.7%) [17]. Industry statistics show the top two places from where nail technicians purchase their nail products are either a professional beauty store or an online‐only distributor [18]. The nail charm products purchased for our study were obtained from both of these sources, demonstrating that nickel‐containing nail products are readily accessible to both nail technicians and consumers.

Given our findings of nickel release from 38.5% of nail charms tested, dermatologists should consider nail charms as a potentially relevant source of nickel contact allergy. Additionally, lead was detected in 2.2% of nail charms. While lead is not a contact allergen, its finding represents a potential exposure to a toxic metal and a potential public safety concern. The presence of lead in nail charm products, applied to the fingernails, is concerning given its known systemic toxicity. If nail art continues to increase in popularity, repeat and more robust investigation into the metal content of nail charm products may be warranted.

## Author Contributions

**Tarah Schaffer:** data curation, writing – review and editing. **Dathan Hamann:** writing – review and editing, supervision. **David Cotter:** writing – review and editing. **Carsten R. Hamann:** conceptualization, methodology, writing – review and editing, supervision. **Brianna C. Peacock:** writing – review and editing. **Emily Ames:** writing – original draft, writing – review and editing, data curation.

## Funding

The authors have nothing to report.

## Ethics Statement

The authors have nothing to report.

## Consent

The authors have nothing to report.

## Conflicts of Interest

Dathan Hamann and Carsten R. Hamann are first degree relatives of an owner of SmartPractice, a company which produces and manufactures patch test supplies/materials. Remaining authors declare no conflicts of interest.

## Data Availability

The data that support the findings of this study are available from the corresponding author upon reasonable request.
